# 

**Published:** 1877-10-20

**Authors:** 


☞ All communications relating to the business of THE RECORD, for the year 1877, must be addressed to

DR. R. C. WORD,

Business Manger Southern Med. Rec.,

Atlanta, Ga.

☞ Brief and practical communications are solicited on all subjects pertaining to medicine, also reports of cases in practice.

☞ Send money by check, postal order or registered letter.

☞ Write your name, post-office, county and State plainly.

### SPECIAL NOTICE.

Parties who receive this number as a specimen copy, are requested to subscribe. We are plain and cheap, but aim to be practical and useful. Try our journal, and see if it does not contain more practical items in less space, and at less cost, than any other journal in this country. May subscribe for six months, though it is best to order the back numbers, and have your volume complete.

☞ A number of communications for our Original Department are crowded out, and will appear hereafter.

☞ Parties in arrears will please settle without further delay. Friends, the year will soon close, and we greatly need the money. Please take due notice thereof, and govern yourselves accordingly.

COLABORATORS AND CONTRIBUTORS.

L. B. Anderson, M.D., Va.

Swan M. Burnett, M.D., D. C

J. M. Bristow M.D., Texas

J. W. Booth, M.D., N C.

W. F. Barr, M.D., Va.

B. W. Corn, M.D., Texas

John Castor, M.D., Iowa.

E. T. Easley, A.M., M.D., Ark

A. H. Goelet, M.D., N. Y.

E C. Gehrung, M.D., Mo.

G. D. Hodge, M.D., Ark.

S. M. Hogan, M.D , Ala.

A. McLane Hamilton, M.D.,NY

C. C. Vanderbeck, M.D., Pa.

Z. B. Herndon, M.D., Va.

Lindsay Johnson, M.D., Ga.

A. R. Kilpatrick, M.D., Tex.

R. Kells, M.D., Miss.

W. L. Lipscomb, M.D., Miss.

J. M. Lewis, M.D., Miss.

G. A. Dyer, M.D., Ind.

C H Lathrop, M.D., Iowa.

A A Lyon, M.D., La

M Lewis, M.D., Tenn

G M Rivers, M.D , S C

A S ayne, M.D., Va.

A. M. Whitehead, M.D., O.

